# Dilemmas in caring for older adults in Zhejiang Province, China: a qualitative study

**DOI:** 10.1186/s12889-019-6637-0

**Published:** 2019-03-15

**Authors:** Sha Ma, Jianwei Shi, Lu Li

**Affiliations:** 1 The Institute of Social and Family Medicine, School of Medicine, Zhejiang University, Room 1001, Comprehensive Building of Medical School, 866 Yuhangtang Rd., Xihu District, Hangzhou, Zhejiang Province 310058 People’s Republic of China; 2 School of Medicine, Tongji University, Shanghai, People’s Republic of China

**Keywords:** Ageing, Old-age care, Community- and home-based care

## Abstract

**Background:**

Owing to the increase in life expectancy and sickliness, caring for older adults has become a major challenge in China, where the traditional care system is disintegrating and community- and home-based care have been introduced to respond to this ‘silver wave’. However, there is limited knowledge of the dilemmas associated with caring for older adults and the acceptability of community- and home-based care for this population.

**Methods:**

Participants were recruited from Xihu District, Hangzhou, from June to July 2017. In-depth interviews were conducted using semi-structured questionnaires. Audio recording, verbatim transcription, and thematic analysis were conducted.

**Results:**

A total of 64 older adults from four communities were interviewed. Half of the participants had multiple chronic diseases. The very old individuals and those with severe diseases and in poor financial conditions were observed to be struggling the most. Health status, financial capability, and personality were the main factors affecting the care process. Participants cited the following reasons for staying away from nursing homes: misunderstanding, negative environment, a sense of shame, loneliness, and financial limitations. Community- and home-based cares are popular forms of old-age care; however, some participants exhibited a lack of knowledge about such services.

**Conclusion:**

A multi-layered old-age care system is urgently needed for older adults in Zhejiang Province. Further, it is important that such a system integrates the care provided through community- and home-based services with that offered by nursing homes. Community- and home-based care for older adults needs to be prioritised, and the quality of care provided in nursing homes should be improved.

**Electronic supplementary material:**

The online version of this article (10.1186/s12889-019-6637-0) contains supplementary material, which is available to authorized users.

## Background

China is experiencing rapid and irreversible population ageing. According to a 2018 report published by China’s Civil Affairs, by 2017, the number of older adults aged above 60 years had reached 241 million, accounting for 17.3% of the total population. Further, a study predicted that, by 2030, 65.6% of the Chinese health burden will be caused by older adults, a population with a high incidence of non-communicable diseases [1]. Caring for older adults has, therefore, been a prominent challenge for China. Generally, care and services for older adults can be divided into formal and informal care. The former refers to old-age care and services provided by nursing homes or private companies. The latter refers to old-age care and services provided by family members, friends, and relatives.

According to China’s social pension system, the government has set up nursing homes to support older adults labelled the ‘Three No’s’, that is, those with no children, no relatives, and no income [2]. Those excluded from the ‘Three No’s’ can live in their homes or nursing homes according to their will and financial capability. Healthy older adults generally manage to perform activities of daily living independently and do not need special care. However, many older adults will eventually become dependent, requiring health services as well as external support [3]. Traditionally, the majority of older adults were cared for by their families, especially when they developed a severe disease or lost the ability to work. It was easy for family members to take care of older adults because several generations lived under one roof [4]. Offspring, particularly sons, were responsible for taking care of older adults until the end of their lives. However, this traditional care system has gradually collapsed [5] owing to the one-child policy [6], low fertility [7], migration wave [6], increase of female employment, and changes in the Chinese filial piety culture. Together, these factors have rendered it less likely for older adults to have the opportunity to live with their adult children. This has forced Chinese older adults to think about their future care needs.

Though all older adults in China face this problem, certain differences have been observed between those living in urban and rural areas. For instance, urban and rural older adults are covered by different welfare policies towing to the urban-rural dual structure in China. The majority of urban older adults are covered by the Urban Residents’ Basic Medical Insurance (URBMI), while rural older adults are covered by the New Rural Cooperative Medical System (NCMS). The former provides higher medical reimbursements for in-hospital and outpatient services pertaining to major diseases as compared to the NCMS. A study showed that rural older adults exhibit lower healthcare utilisation rates as compared to their urban counterparts [8]. Furthermore, these two groups exhibit substantial differences in their financial situation. Specifically, while urban older adults receive retirement pension, rural older adults do not. Further, the majority of urban older adults can choose care services freely, while poor urban older adults can only be provided with limited compensation from the government. Subsidies are only available for the oldest and poorest older adults in rural areas. Consequently, the majority of rural older adults lives on their savings or receives financial support from their children.

Under these circumstances, we have to answer the following questions. Is it feasible for the younger generation to provide care for older adults when several families have only one adult child? Is a nursing home a good choice for older adults when they are incapable of caring for themselves? Are community- and home-based care good choices for older adults? There is an urgent need to answer these questions in the context of mainland China. Thus, the present study aimed to examine the dilemmas associated with caring for older adults; explore the acceptability of nursing homes and community- and home-based care for older adults; and provide reliable suggestions for policy making.

## Methods

### Design

Qualitative interviews were conducted with participants from Hangzhou, Zhejiang Province, China, from 20 June to 28, July 2017. A narrative inquiry was used to guide the interview (Additional file 1). All interviews were conducted by a researcher. The participants’ personal narratives were gathered through the qualitative interviews. Additionally, information on demographic characteristics, health status, attitudes towards and reasons for preferring different types of care, and difficulties experienced in daily life were collected.

### Setting

We interviewed older adults from the communities of Huang gu shan, Zi jin gang, Lan li, and Ji hong in Hangzhou. Huang gu shan and Zi jin gang represent urban areas, while Lan li and Ji hong represent rural areas. Each community comprised thousands of families. In these four communities, certain older adults lived with their family members (such as spouse, son, daughter, son-in-law, daughter-in-law, and grandchildren), while others lived alone. The majority of older adults had retired from their jobs.

### Recruiting participants

**Fig. 1:**
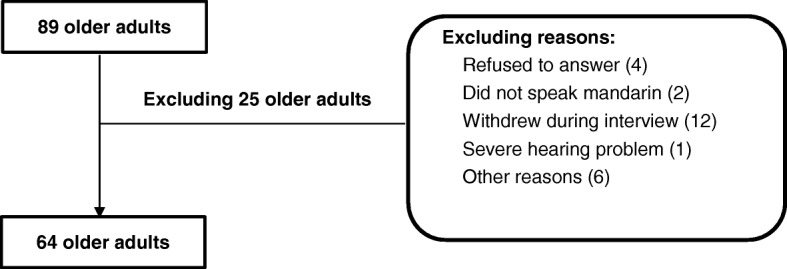
The number of participants and reasons for exclusion

Only community-dwelling older adults aged over 60 years were interviewed in this study. Before conducting the formal interview, the researcher had an informal conversation with the participants. Those with any type of mental illnesses, who refused to participate, or were unable to communicate normally were excluded. A total of 89 older adults from the four communities participated in this study, and finally, 64 of them completed an in-depth interview conducted using a semi-structured questionnaire (Fig. 1).

### Process of investigation

We first contacted community leaders who were responsible for the daily operations of the home care centres in the community. The leaders facilitated opportunities for the older adults to engage in activities such as dancing, watching TV, chatting, and playing poker (or mahjong). Certain communities also conducted simple blood tests periodically. If any of the older adults living alone are unwell, they can contact the community leader for assistance. As these community leaders were liked and trusted by older adults, we arranged for them to make the necessary introduction. They informed the older adults of our intention and asked them to cooperate with us if they wished to participate in the interview. Finally, oral informed consent was obtained from each interviewee and the researcher conducted the interviews, each lasting for 30 to 60 min.

### Data transcription and analysis

The following methods were used in this study: audio recording (30 participants agreed to the recording), verbatim transcription, and thematic analysis. We attempted to record all interviews; however, 34 interviewees refused to be recorded. For such interviews, we only recorded certain key information quickly in our notes and added more detailed information after the interview.

## Results

### Demographic characteristics of participants

In total, 64 participants aged 60 to 88 years were interviewed. Of them, 43 were female and 21 were male; four (6.25%) were from the Huang gu shan community, 11 (17.19%) from the Ji hong community, 11 (17.19%) from the Lan li community, and 38 (59.38%) from the Zi jin gang community (Table 1). Before retirement, the participants had worked in various industries: as teachers, self-employed labourers, civil servants, doctors, policemen, salesperson, workers, cleaners, and farmers. Their financial status varied substantially. Some of them received 7000 CNY as retirement pension per month, while others received no retirement pension. The majority of them reported receiving a retirement pension of 2000 CNY to 5000 CNY per month. Further, 15 (23.44%) older adults were widowed, among whom 7 lived alone, 5 lived with their children, and 3 did not provide details. The remaining 49 older adults were married, among whom 20 lived with their spouses only; 5 lived with their children only; 18 lived with their spouses and other family members such as son/daughter, son-in-law/daughter-in-law, or grandchildren; 1 lived alone; and 5 did not report the necessary information.

**Table 1:** The demographic characteristics of participants

Variables	N	%
Communities		
Huang gu shan	4	6.25
Ji hong	11	17.19
Lan li	11	17.19
Zi jin gang	38	59.38
Gender		
Male	21	32.81
Female	43	67.19
Age		
60–69	19	29.69
70–79	22	34.38
Over 80	23	35.94
Educational level		
Bachelor’s degree	13	20.31
Associate degree	8	12.50
Senior high school	6	9.38
Middle high school	11	17.19
Primary school	11	17.19
Illiterate	12	18.75
Missing information	3	4.69
Occupation		
Cleaner	1	1.56
Dressmaker	1	1.56
Kitchen staff	1	1.56
Electrician	1	1.56
Judge	1	1.56
Sorority director	1	1.56
Self-employed	1	1.56
Engineer	1	1.56
Labourer	11	17.19
Civil servant	8	12.50
Administrator	1	1.56
Accountant	1	1.56
Teacher	11	17.19
Policeman	1	1.56
Farmer	18	28.13
Pharmacist	1	1.56
Salesperson	1	1.56
Missing information	3	4.69

### Health status and needs

Health status was a key factor influencing older adults’ lives. Healthy older adults could freely choose to stay at home or nursing homes because they did not need an external support.

“*I am healthy. I am responsible for cooking, cleaning, and taking care of my grandchildren.”* (HZ-XH-15, male, age bracket: 61-70)

However, the majority of participants, especially the very old individuals, reported having one or multiple diseases, which led to substantial inconvenience. Only 3 (4.69%) older adults were healthy, 27 (42.19%) reported that they were physically healthy in general, 2 (3.13%) had one disease, and 32 (50%) had multiple chronic diseases. These findings imply that the majority of participants, especially those with severe diseases, needed health services and/or daily care (Table 2).

“*I have multiple diseases such as cervical spondylopathy, hypertension, chirobrachialgia, and hearing problems.”* (HZ-XH-01, female, age bracket: 81-90)

*“My health is terrible. I have tracheitis, osphyalgia, and heart disease. I have been taking medicine for two years.”* (HZ-XH-24, male, age bracket: 81-90)

“*I am disabled. She (my wife) helps me do everything because I almost lost my hearing and sight.”* (HZ-XH-50, male, age bracket: 81-90)

**Table 2:** The needs, barriers, and solutions regarding older adults’ care

Needs	Barriers	Caregiver concern	Recommendations
1) Health care services 2) Daily care (such as eating, cleaning, trimming of nails)	1) Lack of informal support 2) Low real purchasing power 3) Incapacity for long-term care 4) Physical deterioration	1) Family members 2) A nurse 3) Nursing home 4) Community and home-based care	Encourage the older adults to live in their homes. Improve the community- and home-care systems. Build and improve the health service and care system. The government should be responsible for the care of older adults, especially the poorest ones.

Healthy older adults or those with mild illnesses often played the role of caregivers in their families. Specifically, six participants reported that they took care of their sick spouses. Nine participants reported that they did all the housework.

### Financial condition and barriers

A potential problem for participants with multiple severe chronic diseases was financial pressure. Prices continue to show an upward tendency, and the participants’ real purchasing power is falling. This could affect their use of health and care services. Further, the majority of older adults with poor health reported that they were experiencing pressure. The frail older adults lacking adequate financial capacity were cared for by their spouses. Further, certain participants reported that, to save money, they self-medicated rather than consulting a physician.

“*My husband needs haemodialysis and renal dialysis. I had been looking after him for a long time and was exhausted. That was when, I hired a temporary worker to help me. We paid her 100 CNY per three hours. However, that was too expensive for us. Finally, I decided to look after my husband by myself.”* (HZ-XH-10, female, age bracket: 71-80)

“*I have multiple chronic diseases, for which I need to take a lot of medication consistently. However, my new rural cooperative health insurance is not applicable here (Hangzhou) and I cannot afford the medicine. So, I asked my son to buy and mail the medicine I need from my hometown, where it is cheaper”* (HZ-XH-59, female, age bracket: 71-80)

### Limitations of daily living

The majority of older adults experienced physical deterioration and difficulty in performing daily activities owing to ophthalmic disease, hearing problems, and functional impairment. Certain older adults with multiple chronic diseases experienced difficulties in ‘simple’ activities such as eating, cleaning, and trimming of nails. Further, healthy older adults’ activities were limited to grocery shopping, cooking, washing, and taking care of their grandchildren.

“*Eating is difficult because I live alone. My son cooks for me, but he cannot always do it… When I visit a restaurant, I prefer to choose foods that are easy to digest…I know the young people dislike my drawling behaviour...Another big problem for me is transfusion. The hospital is too far away, and I am not good at using smartphone apps to book a taxi.”* (HZ-XH-02, female, age bracket: 81-90)

“*I have to take care of my husband, who is completely bedridden. He has not gone downstairs once for the last three years.”* (HZ-XH-48, female, age bracket: 81-90)

“*Life is difficult for a lonely old woman. I cannot do anything in daily life. It is difficult to even trim my nails. I experience pain in my legs and hip joint. I have hypertension, breast cancer, and cataract as well. The doctor said I cannot perform any duties…the older I get, the less labour I should be doing.”* (HZ-XH-35, female, age bracket: 81-90)

### Challenges in daily support from family caregivers

The majority of older adults needed formal or informal support. The former included care services offered by the government, companies, or non-governmental organisations, while the latter included the care provided by family members, friends, and neighbours. Traditionally, intergenerational care was predominant in the Chinese culture, which is characterised by filial piety. However, the majority of the present participants worried about their livelihood when they were incapable or bedridden, especially owing to the lack of caregivers and financial support.

“*It is unrealistic to depend on my son (and daughter-in-law). He already works too hard. His son married a girl who is an only child. My grandson and his wife have to take care of their biological parents and parents-in-law. I do not want to join the ride. My granddaughter-in-law had a baby last year. She has to take care of all of them. Nobody has time to take care of me.”* (HZ-XH-02, female, age bracket: 81-90)

“Children *are too busy. They have to work and take care of their children and grandchildren. Who will care for an old woman like me? ”* (HZ-XH-11, female, age bracket: 71-80)

If family members are no more capable of providing informal care to a fragile older adult owing to the lack of energy and time, is there anybody who can replace them? It may be possible to hire a nurse. However, certain older adults refused to use such services citing the following three reasons: difficulty in finding a good nurse, anxiety about being mistreated, and financial limitations.

“*I have to provide accommodation for them (nurses). There is a possibility that we might have different eating habits. What's more, if she bullies me, I would be unable to fight back.”* (HZ-XH-44, male, age bracket: 81-90)

“*Some nurses are nice, but some are evil. Last year, my mother got sick in Shenzhen. I hired a nurse to take care of her (because I have to stay in Hangzhou to look after my children). The nurse was crafty. My mother always complained on the phone that the nurse was malicious. I did not believe it until I met her.”* (HZ-XH-31, female, age bracket: 61-70)

“I am a common worker. *I receive only 1700 CNY retirement pension a month; it would not even cover the cost of a nurse's meals. How could I hire one? ”* (HZ-XH-45, female, age bracket: 71-80)

### Challenges pertaining to nursing homes

Considering the problems associated with hiring a nurse, is it possible for older adults to live in nursing homes? The present study showed that two determinants, specifically, financial capability and perceptions regarding nursing homes, influenced the older adults’ readiness to go to a nursing home. Those with high financial capacity expressed willingness to live in nursing homes when they were incapacitated because they did not wish to be a burden to their offspring.

“*I will go to a good nursing home that I can afford. Children have no time and energy to look after me. It is reassuring to go to a nursing home because they have professional doctors.”* (HZ-XH-09, female, age bracket: 60-71)

Meanwhile, several participants refused to go to a nursing home when they were incapacitated because they saw it as something shameful.

“*I will not go to a nursing home. I have children. They should take care of me when I am incapacitated. Only those without a child or those with disobedient children should be sent to a nursing home*.” (HZ-XH-16, male, age bracket: 61-70)

“*I will never go to a nursing home. Never.”* (HZ-XH-39, female, age bracket: 61-70)

This revulsion towards nursing homes was observed in both rural and urban older adults. One urban older adult shared an experience and reported the following reasons for his unwillingness to go to a nursing home: negative environment, a sense of shame, and loneliness.

“*I will never go to a nursing home. I visited a retired colleague of mine 10 years ago. All the older adults in the nursing home were cuddled up in a heap (mimicking the action)…I realized that the environment had a negative effect on people's mentality...They were alone…The environment is very important to older adults...My bosom friend was mighty and talkative before he went to that nursing home…Finally, he told me he felt sad and did not want to connect with any acquaintances because he had been abandoned by his children….His children are just too busy actually. The nursing home he stayed at was very upscale. But he still felt sad.”* (HZ-XH-38, male, age bracket: 81-90)

Another important reason why older adults refused to go to a nursing home was financial limitations.

“*I will never go to a nursing home. I have no income (pension) because I am a farmer. Even if my children have money, I do not want to go to a nursing home. I would like to stay in my home.”* (HZ-XH-40, female, age bracket: 71-80)

“*I will never go to a nursing home, even a public one. My retirement pension is 2000 CNY per month, and I cannot afford it.”* (HZ-XH-26, male, age bracket: 71-80)

“*I visited a nursing home a few days ago. A room for two persons was 7000 CNY per month, and that was just the bunk fee. You have to pay extra for food, nursing care (if you need it) and other services...There are cheap ones too; a room for six persons with one toilet costs 3500 CNY per month. However, that is nonsense. How can so many older adults wait to use a toilet in the morning? And I cannot afford it even that...My husband's care was covered by the government. We could live in a good place without any fee. Now that I am alone, I have to pay for everything.”* (HZ-XH-01, female, age bracket: 81-90)

### Awareness regarding community- and home-based care

The Hangzhou government has built several community- and home-based care centres (such as in Zi jin gang, Lan li, and Ji hong) that provide daily services for older adults. However, few older adults were aware of such communities, the community- and home-based care centres need further publicity and development.

“*I know about community- and home-based care, which mainly provides services to unattended older adults. They provide grooming (trimming of nails and massage services) and health services (blood pressure and blood glucose monitoring).*” (HZ-XH-08, male, age bracket: 81-90)

“*I know about community- and home-based care. However, I do not care for it. I do not think need such services until I am 80 years old.”* (HZ-XH-32, female, age bracket: 61-70)

“*I do not know about community- and home-based care. I have no idea about the future...I just hope I can stay at my home when I am incapable of moving around.”* (HZ-XH-37, female, age bracket: 71-80)

“*I do not know about community- and home-based care. I do not know what services they provide to older adults.”* (HZ-XH-40, female, age bracket: 71-80)

**Fig. 2:**
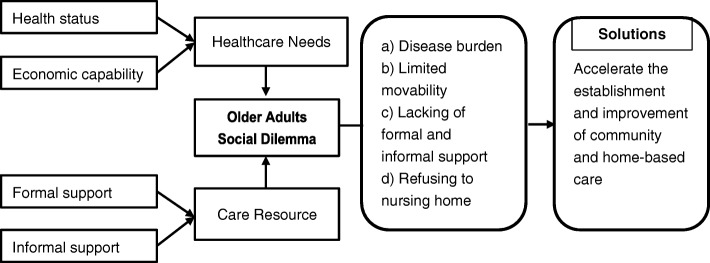
The framework of long-term services and support in China. *Note: Formal support refers to old-age care that involves services provided by nursing homes or private companies. Informal support refers to old-age care that involves services provided by family members, friends, and relatives

Summing up the above findings, dilemmas in caring for older adults arise from the gap between what they need (health status and financial capability) and the actual resources that allow them to access formal and informal support (Fig. 2).

## Discussions

Half of the older adults participating in this study had multiple diseases. A previous study reported that ‘the relationship between physical function and physical activity is bidirectional*’* [9]. The care needs of older adults are predicted to increase rapidly in the coming decades. In the past, the daughters-in-law were responsible for older adults’ care [10] towing to China’s traditional culture and the lower professional participation of females [11]. Nowadays, an increasing number of females have formal jobs, which has weakened the informal care system [12]. Providing support for older adults has become a major challenge, both for the individual and the society. The collapse of traditional family care, combined with insufficient geriatric care resources, has exacerbated the challenges related to old-age care [6]. The present findings are consistent with those of a previous study [13], which reported that the weakening of the social security system, shortage of informal care resources, and older adults’ lack of income affected their access to old-age care and related services.

The majority of participants did not look forward to relying on the younger generation for several reasons. They clearly realized that the younger generation was under a lot of pressure; they were breadwinners and dared not quit their jobs to look after older adults. In addition, some older adults were unwilling to be a burden to their offspring. These findings suggest the urgent need for developing policies to support family care. Further, older adults are often left with two potential choices in response to this plight: hiring a live-in nurse or living in a nursing home. However, these solutions were only suitable for a few open-minded older adults who were in favourable financial conditions. The majority of the present participants refused both options. Lack of trust in nurses was the primary reason for refusing to hire a live-in nurse. Further, most participants were worried about being abused by nurses, the high costs involved, and differences in lifestyle. They refused to go to a nursing home owing to the sense of shame attached to it, financial limitations, negative environment, and poor services. A previous study found that older adults believed that living in a nursing home reflected that their children are impious [14]. Some were worried about being laughed at for living in a nursing home. Others refused to go to a nursing home because of its dull environment. Another previous study reported that nursing home residents tend to lead sedentary lifestyles that lead to adverse outcomes, including decline in physical functioning, lower quality of life, and higher mortality rate [15]. Thus, it is imperative to improve the quality of nursing homes and build a trustworthy system.

The present participants expressed a low willingness to stay in nursing homes, which was consistent with the findings of a previous study [16]. In this study, both residential and migrant older adults (which refer to those who had come to Hangzhou from another city and had lived in Hangzhou for more than 6 months) had a strong will to stay at home and to continue to participate in society. The Chinese healthcare system for older adults is limited, and it only covers those who fulfil the criteria of the ‘Three No’s’ [17]. However, considering the social transformation in China, the lack of caregivers is a serious problem faced by the majority of older adults. Community- and home-based care systems, including informal care provided by family members and formal care provided by paid caregivers, are well developed in Western countries [18]. This has become a basic policy in China as well, in response to the ageing society [19]. According to studies conducted in Beijing [20] and Shanghai [21], such a system is beneficial for older adults in China.

However, the present study revealed that certain older adults did not know about community- and home-based care. Such services face the dual problem of social resistance and imperfect systems [22]. Specifically, they suffer from limited range, lack of resources, low-quality service staff [23] and sole form, understaffing, and inadequate infrastructure [24]. Some are situated in old buildings without elevators, which is inconvenient for older adults and detrimental to their social participation [25]. Thus, the need to improve the quality of care and to contain costs is evident [26]. Further, the Chinese old-age care system erroneously attached great importance to nursing homes, ignoring the potential of community- and home-based care [27]. Therefore, concerted efforts are required to provide high-quality care to older adults.

### Limitations

This study only explored the dilemmas pertaining to old-age care in an ageing society and the related difficulties in Hangzhou. We did not consider other stakeholders’ perspectives, such as those of the younger generation and service providers. In addition, the present results were derived from a small sample because we recruited the participants using non-probability sampling. Therefore, more in-depth research on this topic is recommended.

## Conclusion

Older adults in Zhejiang faced an unprecedented dilemma related to their retirement pension. A multi-layered ageing service system composed of community- and home-based care, nursing homes, and other new forms of services should be established to meet the various needs of older adults. Only a few older adults in favourable financial conditions could afford to live freely in their own homes by hiring a nurse or living in a nursing home and enjoying high-quality services. Therefore, community- and home-based care should be prioritised to cater to the majority of older adults’ needs.

## Additional file

Additional file 1: Semi-structured interview guide. (DOCX 22 kb)

## Abbreviations

NCMS: The New Rural Cooperative Medical System
URBMI: Urban Residents’ Basic Medical Insurance
