# Supplementary material for: Dilemmas in caring for older adults in Zhejiang Province, China: a qualitative study
Source: BMC Public Health. 2019 Mar 15;19:311. doi: 10.1186/s12889-019-6637-0 (PMC6420727; doi:10.1186/s12889-019-6637-0)
Supplement: Supplementary file 1 — Semi-structured interview guide. (DOCX 22 kb) [file 12889_2019_6637_MOESM1_ESM.docx]

**Additional file 1: Semi-structured interview guide**

**I Background information**

(1) Please introduce yourself shortly (Main information include name, age, educational attainment, job before retirement, financial status, marriage and children).

(2) Please describe your relationship with your family members, neighbors, and friends.

**II Health status**

(1) How do you feel about your health?

(2) Do you have any chronic disease? If yes, please describe it.

(3) Is there any problem in your daily life such as grocery shopping, cooking, doing hoursework, doing exercise, and something else?

**III The demand of home-based service**

(1) Please list some urgent services (at least three items) you need based on your health.

(2) Please list some unnecessary services based on your health.

**IV Please offer some information of the home-based services in your community. How do you think about it?**
